# Efficacy and safety of external phytotherapy in diabetic foot ulcers: a GRADE-assessed systematic review and meta-analysis of randomized controlled trials

**DOI:** 10.1186/s13098-025-02049-0

**Published:** 2026-01-09

**Authors:** Xiaodan Yan, Anxin Li, Qian Zhou, Xue Feng, David G. Armstrong, Johnson Boey, Yanzhong Wang, Lu Chen, Wuquan Deng, Qiu Chen

**Affiliations:** 1 Department of Endocrinology and Metabolism, Hospital of Chengdu University of Traditional Chinese Medicine, Chengdu, 610000 China; 2 Department of Endocrinology and Metabolism, School of Medicine, Chongqing University Central Hospital, Chongqing Emergency Medical Centre, Chongqing, 400000 China; 3 Chengdu University of Traditional Chinese Medicine, Chengdu, 610000 China; 4 Department of Surgery, Keck School of Medicine, University of Southern California, Los Angeles, CA 90033 USA; 5 Department of Podiatry, Singhealth Polyclinics, Singapore, Singapore; 6 School of Life Course and Population Sciences, Kings College London, London, SE58AF UK; 7 Health Studies Research, Florence Nightingale Faculty of Nursing, Midwifery & Palliative Care, King’s College London, Methodologies, London, SE58AF UK

**Keywords:** Diabetic foot ulcers, Chinese herbal medicine, Phytotherapy, Meta-Analysis, GRADE approach, Wound healing

## Abstract

**Background:**

Diabetic Foot Ulcers (DFUs) represent a global healthcare challenge, imposing substantial socioeconomic burdens due to their increasing incidence and associated mortality. This study evaluates the efficacy and safety of external phytotherapy (utilizing various plant-derived compounds, including Chinese herbal medicines and plant-derived liposomes, administered topically) for the treatment of DFUs.

**Methods:**

Relevant studies were identified from major electronic databases (PUBMED, EMBASE, WOS, and the Cochrane Library) that were searched up to April 30, 2024. Randomized controlled trials (RCTs) that evaluated the effects of external phytotherapy for DFUs. The treatment group was treated with external phytotherapy plus conventional treatment, while the control group received conventional treatment alone. Two evaluators independently screened and selected literature, extracted data, and assessed the risk of bias. The outcome measures included complete ulcer healing, ulcer improvement, ulcer area reduction, and healing time. Weighted mean difference (WMD), standardized mean difference (SMD), and relative risk (RR) with 95% confidence intervals (CI) were used for data analysis. Heterogeneity was quantified using I² statistics, with appropriate application of fixed-effects or random-effects models. Methodological quality was ensured through Review Manager and Stata software, complemented by GRADE evidence assessment.

**Results:**

Twenty studies with a total of 1,854 participants were identified. Our analysis suggested that compared with conventional treatment, external phytotherapy significantly enhances complete ulcer healing (RR: 1.84; 95% CI: 1.55 to 2.19), promotes ulcer improvement (RR: 1.32; 95% CI: 1.11 to 1.57), reduces ulcer area (WMD: -1.14; 95% CI: -1.45 to -0.83), and accelerates healing time (WMD: -3.93; 95% CI: -7.48 to -0.39). Safety profiles and ulcer depth measurements showed no significant intergroup differences. GRADE assessments indicated high-certainty evidence for most primary outcomes, whereas the evidence for percentage ulcer reduction was of low certainty due to serious inconsistency and imprecision.

**Conclusion:**

External phytotherapy demonstrates potential as an adjunctive treatment for diabetic foot ulcers, improving primary outcomes like complete healing with moderate to high certainty of evidence. Nevertheless, regional bias—with most evidence derived from East Asia—warrants caution in generalizing these results. Further rigorous, multi-regional trials are needed to solidify the evidence base and refine clinical application.

**Supplementary Information:**

The online version contains supplementary material available at 10.1186/s13098-025-02049-0.

## Introduction

The global burden of chronic diseases has shown a consistent upward trend over the past decade, with diabetes cases projected to reach 783 million and associated healthcare costs estimated to surge to $1,054 billion by 2045 [1]. Among diabetes-related complications, Diabetic Foot Ulcers (DFUs) represent a particularly severe and prevalent condition, affecting approximately 19–34% of diabetic patients worldwide [2]. This complex condition arises from multiple contributing factors, including poor glycemic control, microbial infection, peripheral neuropathy, vascular insufficiency, and structural foot abnormalities [3]. DFUs profoundly impact patients’ functional status and quality of life while imposing substantial strain on healthcare systems [4]. Furthermore, impaired wound healing in DFUs often leads to devastating consequences, with amputation rates reaching critical levels and mortality rates ranging from 54% to 79% [5, 6]. The economic impact of DFU management is particularly noteworthy, with treatment costs comparable to those of other major diabetes-related complications [7].

Effective therapeutic interventions are crucial for promoting ulcer healing and reducing amputation and mortality rates in DFU patients [8]. Standard management protocols typically involve glycemic regulation, surgical debridement, and infection prevention [9]. However, conventional treatments face significant limitations, including drug resistance and suboptimal wound healing outcomes, with a 5-year recurrence rate reaching 65% [10]. Emerging evidence suggests that phytotherapy demonstrates therapeutic potential across all phases of diabetic ulcer healing (hemostasis, inflammation, proliferation, and maturation), potentially attributable to its multi-component composition and multi-target mechanisms [11, 12]. This biological plausibility stems from its dual capacity to both mitigate pathological processes (e.g., inflammation and oxidative stress) and activate regenerative mechanisms (e.g., angiogenesis and tissue remodeling), thereby addressing the multifactorial pathogenesis of DFUs [11, 13, 14]. Furthermore, phytotherapeutic approaches may offer enhanced cost-effectiveness and safety profiles compared to conventional treatments [15, 16].

The International Working Group on the Diabetic Foot (IWGDF) 2023 updated guidelines highlight the uncertain therapeutic efficacy of external phytotherapy incorporating Chinese herbal medicine (CHM) for DFUs management [17].Clinical evidence regarding CHM efficacy remains inconsistent, with studies demonstrating divergent outcomes. While some randomized controlled trials reported comparable ulcer healing parameters between herbal and conventional treatments [18–24], others indicated significant therapeutic benefits of phytotherapeutic interventions [25–30].External phytotherapy refers to the therapeutic application of plant-derived bioactive compounds (e.g., Chinese herbal extracts, plant exosomes, or liposomes) via topical formulations such as ointments, hydrogels, patches, or solutions, targeting localized or systemic effects through transdermal absorption. Previous meta-analyses on CHM for DFU have key methodological limitations. Importantly, some analyses have pooled data from both topical and oral routes of administration, preventing a clear evaluation of external phytotherapy [31]. Additionally, most included RCTs showed a high risk of bias (e.g., lacking blinding/allocation concealment), with only 53% of data pooled and insufficient heterogeneity analysis [16].This review addresses these limitations through a more rigorous methodology incorporating GRADE evidence assessment. Given the critical need to address therapeutic uncertainties in DFU management and the growing global burden of this condition, this study investigates the clinical efficacy and safety profile of external phytotherapy as a potential therapeutic intervention. Through rigorous systematic evaluation, we aim to establish an evidence-based foundation for innovative adjuvant treatment strategies, addressing the urgent need for effective DFU management solutions. The exceptional clinical significance of this research lies in its potential to provide definitive evidence regarding phytotherapeutic interventions, which could substantially impact current treatment paradigms and patient outcomes.

## Methods

### Study protocol and registration

This study was conducted in strict accordance with the Cochrane Handbook for Systematic Reviews and PRISMA guidelines (Supplementary file 1) [32, 33]. The research protocol was prospectively registered on the PROSPERO platform. To ensure methodological rigor, we implemented the AMSTAR 2 checklist for comprehensive quality assessment of included studies (Supplementary file 2) [34].

### Search strategy and selection process

Two independent investigators systematically searched four major databases (PubMed, EMBASE, Web of Science, and Cochrane Library) for randomized controlled trials (RCTs) investigating external phytotherapy for DFUS, covering publications up to April 30, 2024. The search strategy incorporated controlled vocabulary and keywords, including: (Chinese medicine OR Chinese herbal medicine OR medicinal plants OR phytotherapy OR botanicals) AND (diabetic foot ulcers OR diabetic foot syndrome OR foot ulcer) AND (clinical trial OR RCT OR randomized). To ensure comprehensive coverage, we supplemented database searches with manual screening of reference lists. Discrepancies between reviewers were resolved through consensus discussions. (Supplementary file 3). Following duplicate removal, we conducted a two-stage screening process: initial title/abstract review followed by full-text evaluation against predefined inclusion criteria for both qualitative synthesis and quantitative meta-analysis.

### Eligibility criteria

The study selection process adhered to PICOS criteria: (1) Population: Adult patients (≥ 18 years) diagnosed with diabetic foot or DFUs; (2) Intervention: Topical application of phytotherapeutic agents, including Chinese herbal medicine or natural plant-based formulations; (3) Comparator: Standard care alone or combined with placebo; (4) Outcomes: Primary endpoints included complete ulcer healing (defined as full epithelialization with complete wound closure) and ulcer improvement (≥ 50% reduction in ulcer area or ≥ 1-grade improvement in Wagner classification). Secondary outcomes comprised ulcer dimensions (area/depth), healing duration, percentage wound reduction, and adverse events; (5) Study design: Randomized controlled trials with accessible complete datasets.

Exclusion criteria encompassed: non-original research (reviews, meta-analyses), preclinical studies (animal experiments, in vitro research), case reports/series, observational studies, trial protocols, and controlled trials lacking appropriate outcome measures or quantitative data for analysis.

### Data collection process and missing data handling

Two independent investigators performed data extraction using standardized forms, capturing the following variables: study identification (first author and publication year), geographic origin, diagnostic criteria, study design characteristics, sample size, intervention details (including treatment protocols, administration methods, and dosage forms), control group parameters, intervention duration, follow-up period, and outcome measures. In instances where endpoint data were unavailable, we extracted data from the time point closest to the study conclusion.

To handle missing data, a tiered approach was employed. First, for irreplaceable key missing data (e.g., unreported outcome data by group), we attempted to contact authors twice; studies were excluded if no response was received. Second, for studies reporting data in alternative formats (e.g., interquartile ranges), we applied prespecified statistical conversions to include them in the meta-analysis, as detailed in Supplementary file 4. Studies with only non-critical missing data were retained, and their impact was assessed via sensitivity analyses.

### Synthesis methods

Statistical analyses were conducted using Review Manager 5.4.1 (Nordic Cochrane Center) and Stata version 17 (Stata Corp). Continuous outcome measures were analyzed using weighted mean difference (WMD) or standardized mean difference (SMD) with 95% confidence intervals (CI). Dichotomous outcomes were evaluated using risk ratios (RR) with 95% CI. We employed fixed-effects models for analyses with low heterogeneity (I²<50%) and random-effects models for those with substantial heterogeneity (I²≥50%) [32]. Study heterogeneity was quantified using I² statistics, categorized as follows: none (I² = 0%), mild (I² ≥ 25%), moderate (I² ≥ 50%), and substantial (I² ≥ 75%).

### Risk of bias and certainty of evidence

Methodological quality was independently assessed by two investigators using the Revised Cochrane Risk of Bias tool (ROB 2) [35]. Each study was judged across the five domains of the tool and assigned an overall risk of bias classification (‘low risk’, ‘some concerns’, or ‘high risk’) according to the tool’s official guidance. An exploratory analysis using a pre-specified quantitative scoring system derived from the ROB 2 judgments was also performed to test the robustness of the findings; the detailed methodology and criteria for this analysis are provided in Supplementary file 5. Publication bias was evaluated through visual inspection of funnel plots, supplemented by Begg’s or Egger’s statistical tests when ≥ 10 studies were available.

Evidence certainty was determined using the Grading of Recommendations Assessment, Development, and Evaluation (GRADE) framework [36]. Any disagreements were resolved through team discussion.

### Subgroup analysis and sensitivity analysis

Subgroup analyses were conducted by study design(double-blind, open-label, unclear), country(China, Iran, Thailand, and others), baseline ulcer grade((included ulcer Wagner grades 1 to 2, included ulcer Wagner grade 3 or higher, unclear), phytomedicine form((solution, ointment, others), and composition(plant Extract, compound), and intervention duration(≤ 8 weeks, > 8 weeks) to explore potential sources of heterogeneity. Sensitivity analyses for each outcome assessed result stability.

## Results

### Study selection and study characteristics

**Fig. 1:**
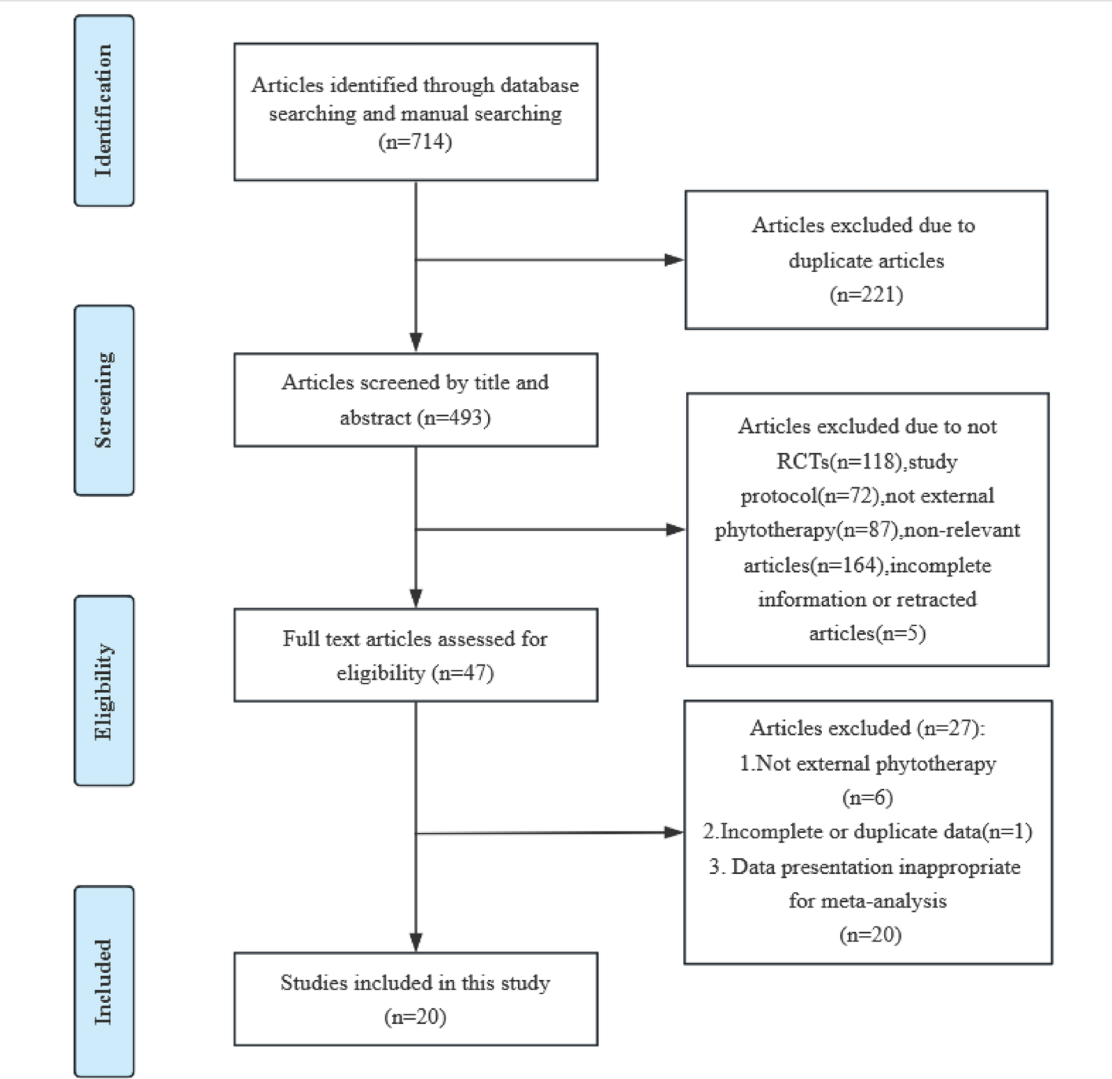
PRISMA flow diagram of study screening and selection

Our systematic search strategy identified 714 potentially relevant studies through database retrieval and manual screening. Following duplicate removal (*n* = 221) and sequential evaluation, we excluded 445 records during title/abstract screening and 27 additional studies after full-text review. The final analysis included 20 randomized controlled trials (RCTs) comprising 1,854 participants (intervention group: *n* = 1,118; control group: *n* = 736), which evaluated the therapeutic efficacy of 17 distinct herbal medicines or botanical extracts for DFUs management [21, 23, 28, 29, 37–51]. Figure 1 presents the complete study selection process by PRISMA guidelines. The detailed table is located at the end of this document(Table 1), which were conducted across Asia and the Americas, including China (*n* = 10), Iran (*n* = 5), Thailand (*n* = 1), Mexico (*n* = 2), United States (*n* = 1), and Brazil (*n* = 1). The intervention groups received various phytotherapeutic formulations, including: L. plantarum culture solution, Quercus infectoria solution, Chitosan-based traditional Chinese medicine composite film, T. polium ointment, Zizhu ointment, ON101 (containing PA-F4 from Plectranthus amboinicus and S1 from Centella asiatica), Q. rubra extract (3% Bensal HP with QRB7), Hongyou Ointment + Shengji Powder, Tangzu Yuyang Ointment, Cortex phellodendri compound fluid (Compound Huangbai liquid), Plantavera gel (Aloe vera/Plantago major), olive oil, A. pichinchensis extract, Dermaheal ointment, YaSP solution, and traditional Chinese medicine fumigation + Shengji Yuhong cream. Control groups received standardized care, including surgical debridement, normal saline solution, standard treatment + Vaseline gauze, standard treatment + placebo, standard wound treatment (local debridement, off-loading, and dressing changes) + saline gauze, Kangfuxin solution, routine care, basic treatment + antimicrobial calcium alginate dressing, standard treatment + hydrogel, basic treatment + silver sulfadiazine cream (micronized or standard), and basic treatment + moist-exposed burn ointment (MEBO). Treatment duration ranged from 2 to 24 weeks across studies.

**Table 1:** Basic characteristics of included studies(This table should be placed at the position mentioned on page7

Study ID	Country	Number(IN, CON)	Gender (Male, Female)	Mean age	Duration of Diabetes (years)	Ulcer Duration(months)	Ulcer grade	Study design	IN (dose, approach)	CON (dose, approach)	Intervention duration	Follow up	Outcomes
Argañaraz Aybar, Julio Nicolás.,2022 [37]	Iran	12,10	16, 6	--	--	--	Unclear	open-label randomized and controlled parallel clinical trial	SUDE + L.plantarum cultures solution	SUDE	12 weeks	--	Adverse events, Microbial Analysis of DFU, Wound Area, Histopathology, Distribution and Numbers of Macrophages in DFUs, Neutrophils Functionality
Chokpaisarn, Julalak.,2020 [38]	Thailand	26, 25	11, 15	57	10.81±7.05	4.88±7.18(IN),4.77±10.83(CON)	Grade 1–2	multicenter, open-label, pre-test, and post-test randomized controlled trial	Quercus infectoria solution, topically applied once a day	Normal saline solution	3 months or until wounds healed completely	--	Wound healing (percentage of wound area reduction, complete wound closure), adverse effects
Du, J.C.,2011 [39]	China	30,30	31, 29	64	10±3.4	--	Grade 1–5	randomized controlled trial	Basic treatment + traditional Chinese medicine composite film	Basic treatment +Vaseline gauze	Until ulcer complete closure or 24 weeks	--	Clinical efficacy (ulcer healing rate), average ulcer healing time
Fallah Huseini, H.,2021 [40]	Iran	29, 26	14,41	58.1±6.4(IN),59.4±7.0(CON)	15.2±7.4(IN), 13.3±7.2(CON)	--	Grade 1–2	double-blind, placebo-controlled, randomized controlled trial	Standard treatment +topical T. polium ointment twice a day	Standard treatment +topical placebo ointment twice a day	4 weeks	8 weeks	Wound Healing Determined, Ulcer Recover, wound healing percentage, Blood Biochemical Test, adverse effects
Fan, W.,2022 [41]	China	44, 42	46, 40	70.95±7.70(IN),71.12±6.52(CON)	--	--	Grade 2–3	randomized controlled trial	Basic treatment+ EPTG+ Zizhu ointment)	medical antibacterial dressing	12 weeks	--	Ulcer healing rate, area, depth, TCM symptom score, visual analogue scale, inflammatory factors, growth factors, adverse events, outcome events
Huang Y Y.,2021 [42]	China	122, 114	175, 61	57.0±10.9	≥10 years(61.0%)	7༎2±13.4	Grade 1–2	randomized controlled, evaluator-blinded phase 3 trial	ON101 applied topically twice daily	Hydrofiber; ConvaTecLtd, applied topically twice daily	10 weeks	16 weeks	Complete healing, Change in WSA, Incidence of patients with 50% reduction, Incidence of wound infection, Ulcer recurrence in WSA, HbA1c, adverse events
Jacobs, A. M.,2008 [43]	America	20, 20	--	--	--	--	Grade 1–2	A blinded study	Bensal HP with QRB7 ointment as adjunctive management	Silver sulfadiazine cream (SSC) as adjunctive management	6 weeks	--	Combined wound diameter, Wound Culture Results, Wound Depth, adverse events
Li, F. L.,2011	China	27, 26	34, 28	54.1 ± 14.8(IN),46.2 ± 13.9(CON)	8 (1–19) (IN), 6 (1–21(CON)	0.72 ± 0.65 years (IN), 0.60 ± 0.41 years (CON)	Unclear	prospective, multi-center, randomized, single-blind, parallel controlled trial	“Hongyou Ointment” +"Shengji Powder” topically applied once a day	Mupirocin ointment, growth factor (bFGF,100 AU/cm^2^), Vaseline topically applied once a day	4 weeks or until wound healing	--	Ulcer Healing Time, the expression levels of β-catenin, c-myc, and K6 proteins, adverse effects
Li, S.,2011	China	24, 24	31,17	60±13	9.8 ± 5.5	≥12 weeks	Grade 1–3	multi-center, randomized, controlled, prospective, add-on clinical trial	SWT + TYO, every one to three days	SWT + saline gauze every one to three days	Until ulcer complete closure or 24 weeks	24 weeks	Complete healing, ulcer improvement
Liu, Y. L.,2020 [28]	China	540, 180	397, 323	63.7±9.56 (IN),63.12±0.53 (CON)	13.21±9.83(IN), 13.54±8.55(CON)	--	Grade 1–2	multicenter clinical trial	CPCF	KSF	4 weeks	short follow-up time	Wound area, Growth factor indexes, primary symptoms, secondary symptoms, treatment efficacy, Adverse events
Najafian, Y.,2019 [29]	Iran	20, 20	28, 12	61.5 ± 7.96(IN),57 ± 8.4(CON)	--	--	Grade 1–2	double-blind randomized clinical trial study	Routine cares + Topical Aloe vera/Plantago major gel (Plantavera gel)	gel	4 weeks	--	Wound surface and depth, degree, color, drainage and surrounding tissues and scaling of the wound, the side effects
Nasiri, M.,2015 [44]	Iran	15,15	19, 11	53.8 ± 1.3(IN), 52.6 ± 9.13 (CON)	12.73 ± 7.48 (IN), 14.93 ± 10.38 (CON)	50.0 ± 28.65 (IN), 44.93 ± 30.37 (CON)	Grade 1–2	double-blind randomized clinical trial study	Routine cares + Topical olive oil	Routine cares	4 weeks	--	Ulcer surface area, Ulcer parameters scores, total ulcer status scores, Ulcer healing status, Adverse effects
Romero-Cerecero, O.,2015 [23]	Mexico	14, 16	17, 19	63±12.33	≥10 years(60%)	-	Grade 1–2	Randomized, Controlled Pilot Study	(5%, cream formulation) of A. pichinchensis	Micronized silver sulfadiazine (1%)	20 weeks-24weeks	-	Wound size reduction (percentage of wound area reduction), average time, lapse required for ulcers to heal, adverse events
Salahi, P.,2024 [45]	Iran	25, 25	36, 14	57.9 ± 9.88(IN),55.6 ± 10.44(CON)	11.0 ± 8.18(IN), 12.8 ± 7.28 (CON)	70.9 ± 89.23 days (IN), 60.8 ± 80.21 days (CON)	Grade 1–2	randomized, placebo-controlled, double‐blinded, parallel-group clinical trial	Standard care + Dermaheal ointment	Standard care + placebo	4 weeks	-	DFU healing checklist (ulcer degree, ulcer color, ulcer peripheral tissues, and ulcer exudates, DFU size, DFU-induced pain severity(a 0–10 Numerical Pain Rating Scale), Adverse effects
Sanpinit, Sineenart.,2024 [46]	Thailand	25, 25	34, 13	55.04 ± 1.87 (IN), 54.48 ± 1.60 (CON)	6.04 ± 0.81 (IN), 10.28 ± 1.34 (CON)	9.99 ± 1.79 months (IN), 4.88 ± 0.73 months (CON)	Grade 1–2	Prospective, multicenter, open-label, randomized, controlled, and parallel-group study	Standard treatment + YaSP solution topically, 2 mL of the oil per cm^2^ of the wound area	Standard treatment	12 weeks	-	Completely healed; Improved ulcer healing; Decreasing in ulcer area orWagner’s ulcer grade
Tonaco, Luís A. B.,2018 [47]	Brazil	27, 23	41,9	-	-	-	Unclear	Pilot Study	Standard treatment +topical dressing formulated with 0.1% P1G10	Standard treatment +hydrogel	16 weeks	-	the initial total ulcer area; adverse effects
Xie, F.,2012 [48]	China	31, 31	45,17	-^a^	-	-	Unclear	Randomized controlled trial	Basic treatment + traditional Chinese medicine fumigation+ Shengji Yuhong cream	Basic treatment	60 days	-	Clinical recovery rate, limb function, wound area, total effective rate, safety
Xu, L.,2023 [49]	China	40, 39	-	59.46±24.89	-	63.41±24.89 days	Grade 1–2	Randomized controlled trial	liquid fomentation once a day	Routine therapy +medical silver nanoparticles containing dressing once a day	6 weeks	-	Ulcer area, ulcer depth, traditional Chinese medicine syndrome score, ABI, transcutaneous oxygen pressure, vascular endothelial growth factor, epidermal growth factor, advanced glycation end product, high-sensitivity C-reactive protein, adverse events
Yang, G.,2024 [50]	China	22, 20	29, 13	66.55 ± 11.959 (IN), 61.55 ± 10.283 (CON)	-	-	Grade 2	double arm study	FFHB + Basic treatment	ACAWD + Basic treatment	2 weeks	1 month	Negative rate of wound culture, change trend of minimum inhibitory concentration, infection control rate, Wound surface area, wound area healing rate, Pharmacoeconomic evaluations (Cost-effect ratio), Wound healing rate, adverse events
Zhan, H.,2021 [51]	China	25, 25	33, 17	65.1 ± 11.7 (IN); 63.4 ± 11.6 (CON)	-	-	Grade 2–4	randomized controlled trial	Basic treatment + moist exposed burn ointment +Jinhuang powder	Basic treatment + moist exposed burn ointment	1 month	-	effective rate, wound pain score

ABI: Ankle-brachial index; ACAWD: Antimicrobial Calcium Alginate Wound Dressing; CON: Control group; CPCF: Cortex phellodendri compound fluid; DFU: Diabetic foot ulcer; FFHB: Fufang Huangbai Fluid; IN: Intervention group; KSF, Kangfuxin solution; NUM, Number; SUDE, surgical debridement; SWT, local debridement of necrotic tissue or callus, off-loading, and dressing changes; TYO: Tangzu Yuyang Ointment

^a^ Data not reported in the original publication, largely due to variations in reporting standards across studies from different time periods and regions

The Hongyou ointment consists of Jiuyi Pellet (Gypsum Fibrosum: hydrargyrum oxydatum crudum = 9:1), Dong Pellet (main ingredient: minium), and Vaseline. Shengji Powder consists of Gypsum Fibrosuum, Resina Draconis, Resina Olibanum, Myrrh, and Borneolum syntheticum. It comprises Huangbai, Lianqiao (Forsythia suspensa), Jinyinhua (Lonicera japonica Thunb), Pugongying (Taraxacum mongolicum Handazz), and Wugong (Scolopendra). ON101 comprises two active pharmaceutical ingredients: PA-F4 from an extract of Plectranthus amboinicus and S1 from an extract of Centella Asiatica. Chinese medicine fumigation consisted of Astragalus 50 g, Sophora 20 g, Safflower 30 g, Angelica 30 g, Chuanxiong 15 g, Phellodendron phellodendron 20 g, Shuanghua 30 g, and Radix Paeoniae 20 g. Zizhu ointment, composed of Astragalus 9 g, comgrass 9 g, cinnabar 9 g, Dragon’s blood 6 g, ejiao 6 g, borneol 3 g) Ruyi Jinhuang powder paste (Beijing Tongrentang, Jinhuang powder, proper amount of vinegar madeinto paste) (composed of trichosanthin, Cortex Phellodendri, rhubarb, turmeric, Angelica dahurica, purple Magnolia officinalis, tangerine peel, liquorice, Atractylodes Rhizoma, and Rhizoma Arisaematis)Bensal HP with QRB7 ointment, (formulation of benzoic acid, 6%; salicylic acid, 3%; and extract of Qrubra,3%)EPTG(Chinese medicine external therapeutic protocol of enriching pus for tissue growth)P1G10 (Latex of Vasconcellea cundinamarcensis)

### Pooled analysis of all studies

#### External phytotherapy group is more effective in complete ulcer healing and improved ulcer condition

**Fig. 2:**
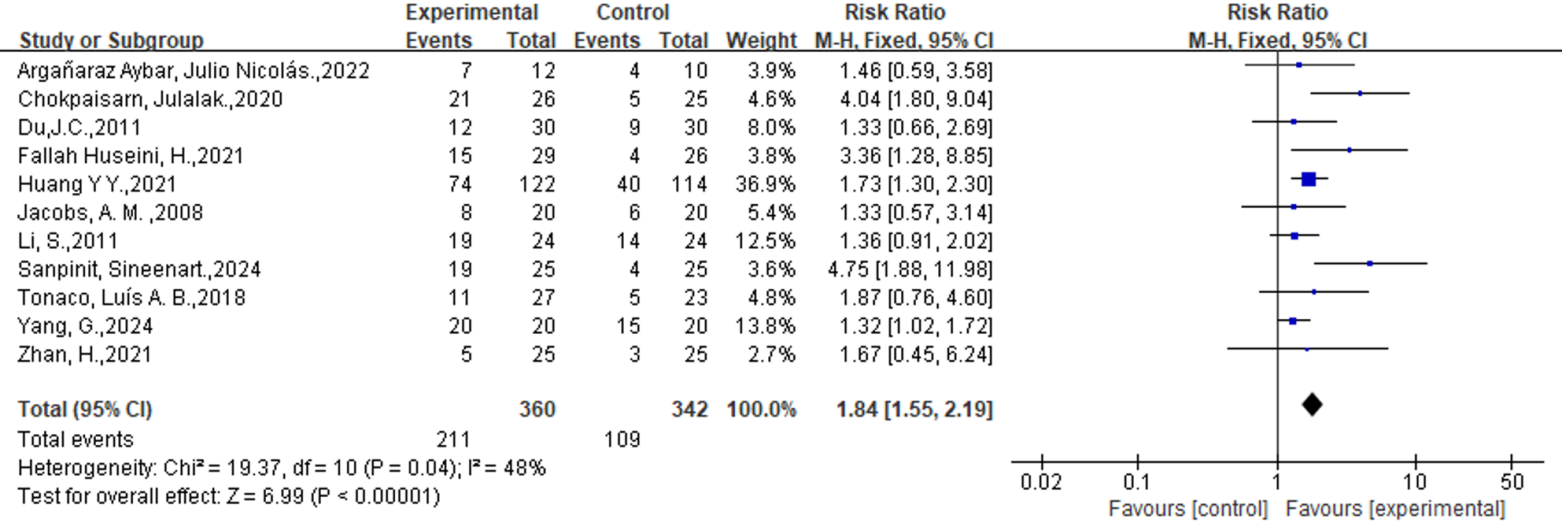
Forest plot of the efficacy of External Phytotherapy on complete ulcer healing

**Fig. 3:**
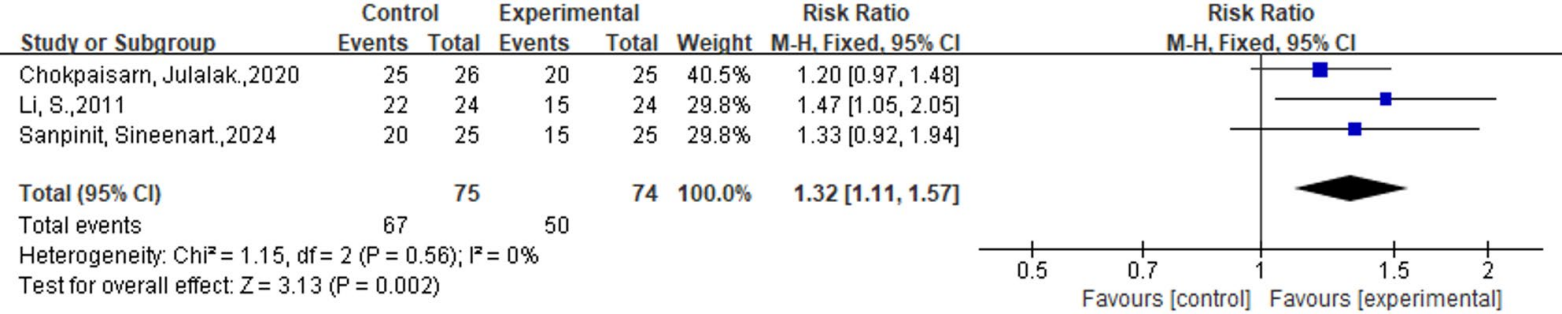
Forest plot of the efficacy of External Phytotherapy on improved ulcer condition

Eleven studies(*n* = 702) reported the incidence of complete ulcer healing, which was higher in the external phytotherapy group than in the control group (58.6% vs. 31.9%; RR: 1.84; 95%CI: [1.55, 2.19]; *p* < 0.00001; I^2^ = 48%) (Fig. 2). Three studies (*n* = 149) assessed the improvement in ulcers. Our analysis showed that the incidence of improved ulcers was higher in the external phytotherapy group (89.3% vs. 67.6%; RR: 1.32; 95%CI: [1.11, 1.57], *p* = 0.002, I^2^ = 0%, Fig. 3).

#### External phytotherapy excels in reducing the area of ulcers

**Fig. 4:**
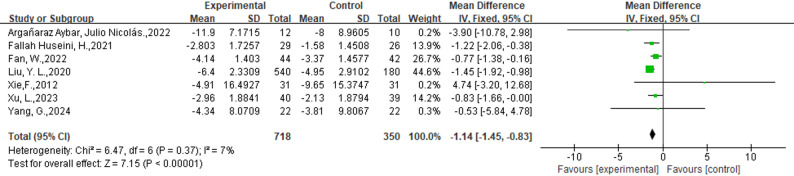
Forest plot of the efficacy of External Phytotherapy on Area of ulcers. The ulcer area is reported in square centimeters (cm²) across all included studies

Seven studies involving 1147 participants (718 in the intervention group and 350 in the control group) were included in the analysis (Fig. 4). Seven studies used L. plantarum culture solution [45], T. polium ointment [40], Zizhu ointment [49], traditional Chinese medicine fumigation [38], and cortex phellodendri compound fluid (three studies) [28, 39, 40]. We observed a significant decrease in the ulcer area in the intervention group (WMD: −1.14; 95%CI: [−1.45, −0.83], *p* < 0.00001, I^2^ = 7%).

#### External phytotherapy did not differ from the control group in ulcer depth

**Fig. 5:**
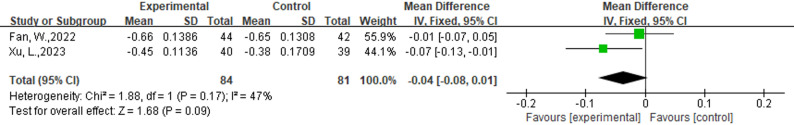
Forest plot of the efficacy of External Phytotherapy on ulcer depth

Ulcer depth was assessed in two studies, with 84 and 81 cases in the intervention and control groups, respectively (Fig. 5). Statistical analysis revealed no statistically significant difference in ulcer area change between the intervention and control groups (WMD: −0.04; 95%CI: [−0.08, 0.01], *p* = 0.09, I^2^ = 47%).

#### External phytotherapy outperformed in percentage reduction in ulcers

**Fig. 6:**
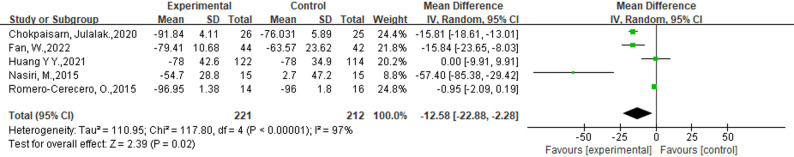
Forest plot of the efficacy of External Phytotherapy on Percentage reduction in ulcers

Five studies reported a percentage reduction in ulcers, with 221 and 212 participants allocated to the intervention and control groups, respectively (Fig. 6). Four studies demonstrated that external phytotherapy and standardized treatment reduced the percentage of ulcer area [23, 38, 41, 42]. However, one study revealed a decrease of 54.7% in ulcer areas in the intervention group and an increase of 2.7% in the control group [50]. The meta-analysis results showed a statistically significant difference between the intervention and control groups regarding percentage reduction in ulcers (WMD: −12.58; 95%CI: [−22.88, −2.28], *p* = 0.02, I^2^ = 97%). Given the high heterogeneity among the studies, we conducted a subgroup analysis to explore the sources of heterogeneity, but no meaningful conclusions were obtained.

#### External phytotherapy shortened the ulcer healing time

**Fig. 7:**
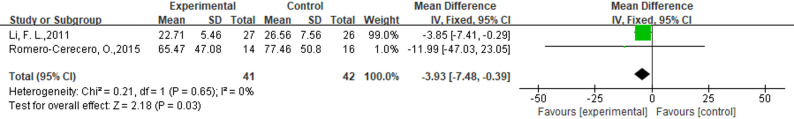
Forest plot of the efficacy of External Phytotherapy on ulcer healing time

Two studies with 83 participants reported data on the ulcer healing time (Fig. 7). The analysis revealed a statistically significant reduction in ulcer healing time in the external phytotherapy group compared to that in the control group (WMD: −3.93; 95%CI: [−7.48, −0.39], *p* = 0.03, I^2^ = 0%).

### Adverse events

**Fig. 8:**
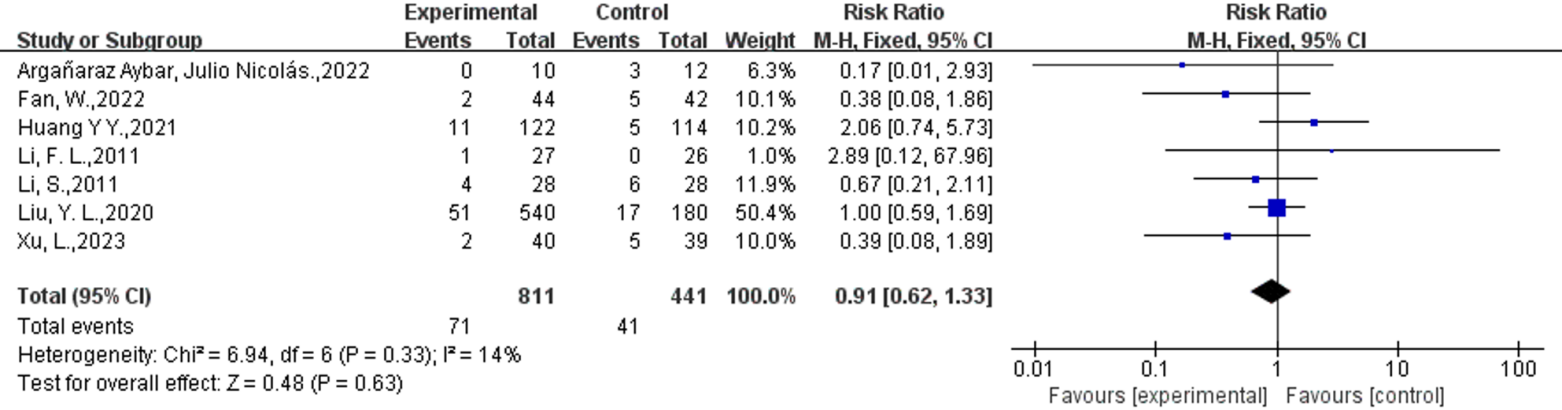
Forest plot of the adverse events of External Phytotherapy

Safety outcomes were reported in 16 RCTs, with nine studies indicating no adverse events in the intervention and control groups [23, 29, 38, 40, 43–45, 47, 50]. Among the remaining seven studies (intervention group: *n* = 811; control group: *n* = 441), adverse events were documented (Fig. 8) [13, 21, 28, 37, 41, 42, 49]. Four studies documented controllable adverse events, such as manageable pain, hypoglycemia, itching, and bleeding on the ulcer surface [13, 28, 37, 41, 42, 49]. Li S did not provide details regarding adverse events [21]. Huang Y Y reported that a single serious adverse event (death of septic shock, acute kidney injury, and acute respiratory failure) occurred associated with the research in the control group, which was considered unrelated to the treatment of ulcers [42]. The meta-analysis showed no significant difference in the occurrence of adverse events between the intervention and control groups (8.8% vs. 9.3%; RR: 0.91; 95%CI: [0.62,1.33], *p* = 0.63, I^2^ = 14%). The safety aspect suggests that no substantial disparity exists between external phytotherapy and standardized treatment methods.

### Risk of bias and publication bias

The results of the comprehensive bias risk assessment indicated that six studies were classified as low risk [23, 29, 40, 43, 45, 47], eleven studies as Some concerns [21, 28, 37–39, 41, 42, 45, 49–51], three studies were identified as high-risk [13, 44, 48](Table 2). Regarding the randomization process, two studies were identified as having a high risk because of an inappropriate randomization method [44, 48]. Concerning deviations from intended interventions, one study was identified as having a high risk due to open-label and a high number of losses to follow-up [13]; the remaining open-label studies were considered to have some concerns [21, 28, 37–39, 41, 42]. As for the measurement of the outcome, one study was identified as having a high risk because of the possibility that outcome measures were not blinded [48]. Moreover, all studies were identified as having a low risk of missing outcome data or selecting reported results.

**Table 2:** Risk of bias for each study

Study ID	Randomization process	Deviations from intended interventions	Missing outcome data	Measurement of the outcome	Selection of the reported result	Overall risk of bias
Argañaraz Aybar, Julio Nicolás.,2022 [37]	Low	Some concerns	Low	Some concerns	Low	Some concerns
Chokpaisarn, Julalak.,2020 [38]	Low	Some concerns	Low	Low	Low	Some concerns
Du, J.C.,2011 [39]	Some concerns	Some concerns	Low	Some concerns	Low	Some concerns
Fallah Huseini, H.,2021 [40]	Low	Low	Low	Low	Low	Low
Fan, W.,2022 [41]	Low	Some concerns	Low	Low	Low	Some concerns
Huang, YY.,2021 [42]	Low	Some concerns	Low	Low	Low	Some concerns
Jacobs, A. M.,2008 [43]	Low	Low	Low	Low	Low	Low
Li, F. L.,2011	Low	High	Low	Low	Low	High
Li, S.,2011	Low	Some concerns	Low	Low	Low	Some concerns
Liu, Y. L.,2020 [28]	Low	Some concerns	Low	Low	Low	Some concerns
Najafian, Y.,2019 [29]	Low	Low	Low	Low	Low	Low
Nasiri, M.,2015 [44]	High	Low	Low	Low	Low	High
Romero-Cerecero, O.,2015 [23]	Low	Low	Low	Low	Low	Low
Salahi, P.,2024 [45]	Low	Low	Low	Low	Low	Low
Sanpinit, Sineenart.,2024 [46]	Some concerns	Some concerns	Low	Low	Low	Some concerns
Tonaco, Luís A. B.,2018 [47]	Low	Low	Low	Low	Low	Low
Xie, F,2012 [48]	High	Some concerns	Low	High	Low	High
Xu, L.,2023 [49]	Some concerns	Some concerns	Low	Low	Low	Some concerns
Yang, G.,2024 [50]	Low	Some concerns	Low	Low	Low	Some concerns
Zhan, H.,2021 [51]	Some concerns	Some concerns	Low	Low	Low	Some concerns

Publication bias was assessed using Egger’s test for studies reporting complete ulcer healing, demonstrating no significant bias (*p* = 0.103). Visual inspection of funnel plots revealed symmetrical distributions for all outcomes except “percentage reduction in ulcers,” suggesting minimal publication bias for most endpoints (Supplementary file 6).

### Subgroup analysis

According to the Wagner grading system, foot ulcers are classified from level 0 to level 5, with level 3 or higher indicating the presence of infection [52]. We performed subgroup analyses for complete ulcer healing, area of ulcers, and adverse events by study design (double-blind, open-label, unclear), country (China, Iran, Thailand, and others), baseline ulcer grade (included ulcer Wagner grades 1 to 2, included ulcer Wagner grade 3 or higher, unclear), phytomedicine form (solution, ointment, others), phytomedicine composition (plant Extract, compound), and intervention duration (≤ 8 weeks, > 8 weeks). None of the subgroups in the area of ulcers or adverse events exhibited statistically notable disparities between the intervention and control cohorts.*The* outcomes of the subgroup analysis for complete ulcer healing are presented in Table 3. Three key findings emerged from subgroup analysis of complete ulcer healing: (1) Geographical disparity: Iranian/Thai studies showed significantly higher healing rates than Chinese studies (RR = 2.41 vs. 1.52; *P* = 0.01). (2) Plant extract superiority: Mono-herbal extracts outperformed compound formulations (RR = 2.13 vs. 1.36; *P* = 0.009). (3) Consistent performance: No significant differences across study designs, ulcer grades, or treatment durations (*P* > 0.05).

**Table 3:** Subgroup analysis for complete ulcer healing

Complete ulcer healing		Num of trials	Sample size (IN, CON)	Effect size (RR)	95% CI	I-squared (%)	*P* for heterogeneity	I-squared between subgroup (%)	*P* for between subgroup heterogeneity
Study design	Double-blind	2	56,49	2.53	[1.31,4.86]	0	0.0006	19.2	0.29
	Open-label	7	255,244	1.85	[1.52,2.25]	62	<0.0001		
	Unclear	2	49,49	1.41	[0.94,2.12]	0	0.1		
**Country**	China	5	231,213	1.54	[1.28,1.87]	0	<0.00001	73.1	0.01*
	Iran	2	86,81	2.39	[1.23, 4.66]	39	0.01		
	Thailand	2	51,50	4.35	[2.37,7.99]	0	<0.00001		
	Others	2	47,43	1.59	[0.85,2.96]	0	0.14		
Baseline ulcer grade	Included ulcer Wagner grades 1 to 2	5	222,210	2.52	[1.54, 4.13]	58	0.0003	60.5	0.08
	Included ulcer Wagner grade 3 or higher	4	91,89	1.38	[1.01, 1.89]	0	0.04		
	Unclear	2	47,43	1.36	[1.06, 1.75]	0	0.02		
Phytomedicine form	Solution	4	83,80	2.38	[0.95, 5.98]	86	0.06	26.1	0.26
	Ointment	3	73,70	2.07	[1.08, 3.95]	42	0.004		
	Others	4	204,192	1.66	[1.03, 2.68]	0	<0.0001		
**Phytomedicine composition**	Extract	7	261,243	2.13	[1.70, 2.66]	40	<0.00001	85.4	0.009*
	Compound	4	99,99	1.36	[1.06, 1.74]	0	0.01		
Duration ^a^	≤ 8 weeks	5	124,121	1.58	[1.18, 2.12]	11	0.002	31	0.23
	>8 weeks	6	236,221	1.97	[1.60, 2.44]	0	<0.00001		

CON: Control group; IN: Intervention group; Num: Number

Weights were obtained using the random-effects model. The horizontal lines represent 95% confidence intervals

a. Duration refers to the time from baseline until complete ulcer healing rate data are available

* *P* < 0.05 for subgroup difference

Bold = Statistically significant subgroup

Subgroup analyses revealed several noteworthy findings. First, no significant differences were observed between double-blind and open-label studies or across subgroups stratified by phytomedicine formulation or baseline ulcer severity (*p* > 0.05), suggesting consistent therapeutic efficacy of topical phytotherapeutic agents across different administration forms and disease stages. Interestingly, intervention duration did not significantly influence outcomes, potentially reflecting inherent study heterogeneity and variable follow-up periods rather than true therapeutic effects.

Geographical subgroup analysis revealed statistically significant variations in complete ulcer healing rates across different regions (Pfor subgroup difference = 0.01). Studies conducted in Iran and Thailand demonstrated markedly higher healing rates compared to those from China and other regions. The reasons for these disparities are unclear but could hypothetically involve regional differences in formulation or healthcare delivery; however, this remains speculative pending future investigation. These notable disparities prompt consideration of underlying factors such as potential differences in healthcare delivery systems, the standardization and application of phytotherapeutic interventions, and unmeasured population characteristics. However, these findings should be interpreted with caution due to the substantial clinical heterogeneity and the limited number of studies within each regional subgroup, which precludes definitive conclusions.

Comparative analysis of herbal medicine composition revealed superior efficacy of plant extracts over compound preparations (*p* = 0.009), potentially attributable to higher concentrations of bioactive compounds, more standardized extraction protocols, and reduced variability in individual responses observed with single-plant formulations.

### Sensitivity analysis

Sensitivity analysis was conducted using two approaches to assess the robustness of our findings. First, the leave-one-out method demonstrated consistent results for complete ulcer healing, ulcer area, and adverse events, with no single study disproportionately influencing the overall pooled estimates. Second, to specifically address the potential impact of studies with high risk of bias, we excluded trials rated as ‘High’ risk (Xie 2012, Li FL 2011, Nasiri 2015). The pooled estimates for complete ulcer healing (RR = 1.85, 95% CI: 1.52–2.26), ulcer area (SMD = − 1.01, 95% CI: − 1.30 to − 0.72), and adverse events (RD = − 0.02, 95% CI: − 0.05 to 0.01) remained statistically significant and directionally consistent, indicating minimal influence of these high-bias studies on the primary conclusions.

In contrast, the outcome of percentage ulcer reduction demonstrated marked vulnerability. This outcome lost statistical significance upon the exclusion of specific studies (e.g., *p* = 0.06 upon excluding Chokpaisarn; *p* = 0.11 upon excluding Julalak or Nasiri) in the leave-one-out analysis. Similarly, in the high-bias exclusion analysis, significance was lost (*p* = 0.11) with persistence of high heterogeneity (I² = 95%) after excluding Nasiri and Chokpaisarn. This indicates that the statistical significance of the percentage ulcer reduction outcome is dependent on the inclusion of these specific studies, rendering the evidence fragile. Consequently, these findings warrant cautious interpretation, a limitation that is directly reflected in the downgraded certainty of evidence for this outcome in the GRADE assessment (Sect. "Certainty of the Evidence: GRADE Assessment").

### Certainty of the evidence: GRADE assessment

GRADE assessment results indicated high-quality evidence for complete ulcer healing, ulcer area changes, depth measurements, healing duration, and adverse events. Two levels downgraded evidence certainty for the percentage ulcer reduction due to serious inconsistency and imprecision. This decision was based on the sensitivity analysis (Sect. "Sensitivity analysis"), which showed a loss of statistical significance (*p* = 0.11) upon exclusion of high-risk studies, alongside persistently high heterogeneity (I²=97%).This indicates limitations in current evidence to explain variability in treatment effects(Table 4).

**Table 4:** Results of the grading assessment for each outcome

Outcomes	No of studies	Study design	Risk of bias	Inconsistency	Indirectness	Imprecision	Other considerations	External Phyto -therapy	standard treatment	Relative (95% CI)	Absolute (95% CI)	Certainty
Complete ulcer healing	13	randomized trials	not serious	not serious	not serious	not serious	none	211/360 (58.6%)	109/342 (31.9%)	RR 1.84 (1.55 to 2.19)	268 more per 1,000 (from 175 more to 379 more)	⨁⨁⨁⨁ High
Improved ulcer	3	randomized trials	not serious	not serious	not serious	not serious	none	67/75 (89.3%)	50/74 (67.6%)	RR 1.32 (1.11 to 1.57)	216 more per 1,000 (from 74 more to 385 more)	⨁⨁⨁⨁ High
Area of ulcers	7	randomized trials	not serious	not serious	not serious	not serious	none	718	350	-	MD 1.14 lower (1.45 lower to 0.83 lower)	⨁⨁⨁⨁ High
Depth of ulcers	2	randomized trials	not serious	not serious	not serious	not serious	none	84	81	-	MD 0.04 lower (0.08 lower to 0.01 higher)	⨁⨁⨁⨁ High
Percentage reduction in ulcer	5	randomized trials	not serious	Serious^a^	not serious	Serious^b^	none	221	212	-	MD 12.58 lower (22.88 lower to 2.28 lower)	⨁⨁◯◯ Low
Ulcer healing time	2	randomized trials	not serious	not serious	not serious	not serious	none	41	42	-	MD 3.93 lower (7.48 lower to 0.39 lower)	⨁⨁⨁⨁ High
Adverse events	7	randomized trials	not serious	not serious	not serious	not serious	none	71/811 (8.8%)	41/441 (9.3%)	RR 0.91 (0.62 to 1.33)	8 fewer per 1,000 (from 35 fewer to 31 more)	⨁⨁⨁⨁ High

The table presents the results of the grading assessment for each outcome using the grading tool. The grading assessment evaluates the quality of evidence and strength of recommendations for the outcomes analyzed in the meta-analysis. ⊕⊕⊕◯: Low Certainty; ⊕⊕◯◯༚Moderate Certainty; ⊕⊕⊕⊕༚High Certainty; CI: confidence interval; MD: mean difference; RR: Risk ratio

Explanations: a. The analysis exhibited extreme heterogeneity (I² = 97%). Pre-specified subgroup analyses (by plant type and extraction method) and sensitivity analyses failed to resolve the sources of this heterogeneity, indicating unexplained variability in effect estimates across studies b. The finding lost statistical significance (*p* = 0.11) upon exclusion of the studies by Nasiri and Chokpaisarn in the sensitivity analysis, indicating fragility. Moreover, the 95% confidence interval of the pooled estimate is sufficiently wide to encompass both a clinically significant benefit and a null effect. I²=97% with unresolved heterogeneity sources after Subgroup analysis (plant type/extraction method) and Sensitivity analysis (sequential exclusion)

## Discussion

### Summary of evidence

Our comprehensive evaluation of external phytotherapy for DFUs management examined six critical outcome measures: complete ulcer healing, ulcer improvement, ulcer area reduction, depth measurement, percentage area reduction, and healing duration. The meta-analysis of topical phytotherapeutic applications, including single-plant extracts and compound formulations, demonstrated superior efficacy compared to standard care, particularly in complete healing rates (RR: 1.84; 95% CI: 1.55–2.19), ulcer improvement (RR: 1.32; 95% CI: 1.11–1.57), and wound area reduction (WMD: −1.14; 95% CI: −1.45 to −0.83). Notably, phytotherapy showed potential to accelerate healing duration (WMD: −3.93 days; 95% CI: −7.48 to −0.39), suggesting its value as an adjunctive treatment modality.

Safety analysis revealed comparable adverse event rates between phytotherapy and standard treatment groups (8.8% vs. 9.3%; RR: 0.91; 95% CI: 0.62–1.33), indicating that topical herbal applications do not increase treatment-related risks.

When contextualized within the existing literature, this study provides significant methodological and clinical advancements over previous meta-analyses in four key aspects:

(1) Broader Geographical Representation: While Wang et al. [16]included exclusively Chinese trials and Zamanifard et al. [31]had limited generalizability despite no country restrictions, our analysis incorporated studies from 11 countries (e.g., Thailand, Iran), thereby improving the global applicability of the findings.

(2)Stratified Analysis to Address Heterogeneity: We implemented pre-specified subgroup analyses to address clinical heterogeneity. This approach allowed for the quantitative synthesis of 85% (17/20) of the included RCTs, providing a more nuanced understanding of how clinical factors influence the treatment effect.​​.

(3) Quantitative Safety Assessment: Building on previous reviews that offered qualitative safety summaries [16] or focused on specific agents [31], our analysis expands the evidence base by establishing a comprehensive, quantitative safety profile for topical phytotherapy (RD = − 0.02, 95% CI: − 0.05 to 0.01).

(4) Exploration of Intervention Types: Our analysis examined the effect of intervention type, a comparison not detailed in earlier reviews [16, 31]. The results indicated that plant extracts were associated with a higher rate of ulcer healing than compound formulations (RR = 2.13 vs. 1.36; *p* = 0.009).

Collectively, these findings contribute to a more robust evidence base, which can help refine clinical decision-making and guide future research directions. Through its methodological approach, this study strengthens the understanding of phytotherapy’s role in DFUS management.

### Possible mechanism

The pathogenesis of DFUs is strongly linked to hyperglycemia [9, 53, 54]. Hyperglycemia can induce peripheral nerve and vascular damage, oxidative stress, inflammation, and impaired skin barrier function [53, 55–58] while also being influenced by trauma, prolonged sitting, psychological stress, smoking, alcohol consumption, and foot deformity [10, 11, 59–62], thereby further promoting ulcer formation.

**Fig. 9:**
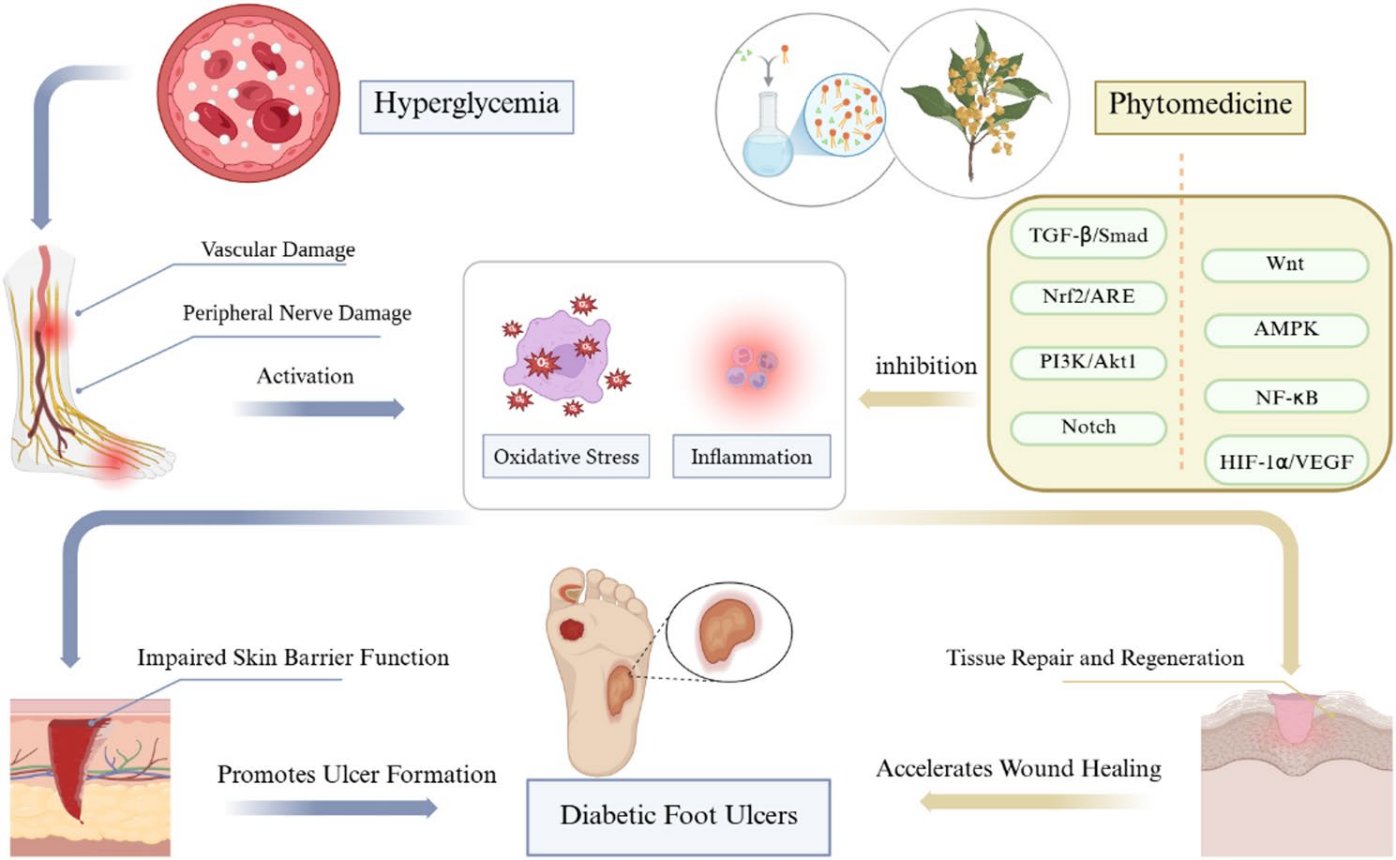
Pathogenesis and Therapeutic Mechanisms of Diabetic Foot Ulcers with External Phytotherapy. Left: Hyperglycemia-induced pathological cascade (vascular damage, oxidative stress, inflammation). Right: Therapeutic actions of phytomedicine through pathway modulation (e.g., TGF-β/Smad for tissue repair). Icons: Blood vessel (vascular damage), ROS symbol (oxidative stress), inflammasome (inflammation), plant leaf (phytotherapy)

The active components of external phytotherapy can enhance foot ulcer recovery by reducing inflammation, fighting bacteria, reducing oxidative stress, preventing cell death, regulating glucose metabolism, promoting tissue repair and regeneration, and stimulating blood vessel formation [13, 14, 63–67]. The active constituents present in plants exhibit synergistic therapeutic effects through multiple pathways, including the TGF-β/Smad, Wnt, Nrf2/ARE, AMPK, PI3K/Akt1, NF-κB, Notch, and HIF-1α/VEGF [11, 13, 14, 68, 69]. Li et al. investigated the potential correlation of applying Hongyou Ointment and Shengji Powder to enhance healing in DFUs through the Wnt pathway [13]. Chebulae Fructus Immaturus extract promotes wound healing in diabetic wounds via activation of the PI3K/AKT and HIF-1α pathways [66]. The primary active component of Astragalus, Calycosin-7-glycoside, may treat DFUs by enhancing the recruitment of anti-inflammatory monocytes, reducing mitochondrial glycolysis, and triggering M2 macrophage polarization through the ROS/AMPK/STAT6 pathways [63]. Sun et al. found that aqueous Gynura divaricata treatment of DFUs is possible with AKT1, TP53, IL6, CASP3(caspase-3), TNF, and VEGFA [67]. Jinhuang Powder promotes wound healing by regulating cell growth, differentiation, and programmed cell death, potentially influencing insulin resistance [14]. Liu Y et al. studied the mechanisms of wound healing in diabetic ulcers in five plants, Bauhinia purpurea, Paeoniae rubrae, Angelica dahurica, Acorus calamus L, and Radix Angelicae biseratae, by proteomic and transcriptomic analyses. They found that the plant extracts promote angiogenesis, increase the proliferation of M2 macrophages, activate key signalling pathways, and regulate the expression of key proteins and miR-s [70]. Another study conducted through network pharmacology and in vitro experiments found that Schisandra chinensis, especially its main component, Gomisin A, effectively promotes wound healing by positively regulating the Phosphorus metabolic process and positively regulating cell migration [71]. The latest studies suggest that potential targets for therapeutic intervention may include macrophage polarization, D- methylation, demethylation, and reactive oxygen species [14, 42, 72–76]. Considering its anti-inflammatory and antibacterial effects, external phytotherapy may enhance the treatment of DFUs coinfection. However, further studies are required to confirm these findings. A schematic diagram elucidating the pathogenesis of diabetic foot ulcers and the therapeutic mechanisms of external phytotherapy has been constructed (Fig. 9). Figure 9 schematically summarizes the key concepts discussed in this section. It illustrates how hyperglycemia(left panel) drives diabetic foot ulcer pathogenesis through vascular/nerve damage, oxidative stress, and inflammation, ultimately impairing skin barrier function. Conversely, phytomedicine(right panel) counteracts these processes by modulating critical signaling pathways (e.g., TGF-β/Smad, PI3K/Akt) to reduce oxidative stress/inflammation and promote tissue repair.

### Strengths

This systematic review represents the first comprehensive evaluation of external phytotherapy versus standard monotherapy for DFUs, offering several methodological and clinical advancements: (1) Inclusion of exclusively randomized controlled trials with rigorous design, demonstrating low risk in key methodological domains including randomization, allocation concealment, blinded outcome assessment, and intention-to-treat analysis. (2) The international scope encompassing 20 studies from diverse geographical regions (China, Iran, Thailand, the United States, Brazil, and Mexico) and ethnic populations, enhancing the generalizability and clinical applicability of findings. (3) Large-scale analysis incorporating 1,854 DFU patients across Wagner grades 1–5, evaluating various phytotherapeutic formulations (extracts and compound preparations) in multiple delivery forms (ointments, solutions, and gels), ensuring broad clinical representativeness. (4) Implementation of robust quality assessment protocols, including methodological evaluation using the ROB2 tool, which indicated a low-to-moderate risk of bias across most studies, and evidence grading through the GRADE framework to establish outcome-specific evidence reliability.

### Limitations

While our findings provide valuable insights, several limitations warrant consideration: (1) The open-label design of some studies may introduce potential bias, although subgroup analysis revealed no significant differences between double-masked and open-label trials (*p* > 0.05). The inherent characteristics of phytotherapeutic interventions (distinct colors and odors) present challenges for placebo-controlled designs. We mitigated this limitation through blinded outcome assessment to enhance result reliability. In our GRADE assessment, the performance bias risk from open-label designs was considered. For the objective outcome of ulcer healing, we judged that downgrading was not warranted, as blinded outcome assessment was employed and subgroup analysis showed no differential effects. (2) Substantial heterogeneity was observed (I² >50% for key outcomes), potentially attributable to variations in study design and intervention protocols. The limited number of studies available for each outcome further restricted more advanced statistical explorations of heterogeneity, such as meta-regression, which precludes a more nuanced understanding of the contribution of specific covariates to the observed heterogeneity. While subgroup and sensitivity analyses confirmed overall robustness, the sources of heterogeneity remain unclear. Notably, for the outcome of percentage ulcer reduction, the high heterogeneity and its instability, as evidenced by the sensitivity analysis (e.g., loss of significance upon exclusion of specific studies), directly led to a downgrading in the certainty of evidence in our GRADE assessment, thereby limiting the strength of any conclusions that can be drawn from this particular outcome. Future meta-analyses should employ bias-adjusted models when high-risk studies contribute substantial weight. (3) Participant distribution was uneven, with two studies contributing a substantial proportion of the total sample size, while most remaining studies had limited sample sizes (≤ 30 participants per group), potentially affecting result generalizability and patient diversity representation. (4) Variable follow-up durations across studies may limit the assessment of long-term efficacy and safety profiles, as shorter observation periods might not capture all relevant clinical outcomes. (5) Crucially, key outcomes including ulcer depth and healing time were reported in only two studies, which precludes definitive conclusions due to severely limited evidence. (6) Heterogeneity and non-standardized reporting of key parameters: Substantial variations were observed in the reporting of intervention dosage (types of plants, formulations, regimens) and adverse events. Critically, adverse events like pain or itching were typically reported only as present/absent or with qualitative descriptors, lacking standardized severity scales (e.g., VAS). This, coupled with inconsistent dosage parameter reporting, precluded meaningful subgroup or dose-response analyses for dosage, as well as any quantitative pooled analysis of adverse event severity. Future research must prioritize standardization in reporting these key parameters. (7) The significant geographical heterogeneity (manifested as superior efficacy in Iran/Thailand) warrants cautious interpretation. Current evidence suggests potential contributions from: healthcare system disparities, formulation-specific attributes, and population characteristics, though limited reporting prevents definitive conclusions. These regional differences should therefore be framed as hypotheses-generating for future studies. (8) Limited external validity: The generalizability of our findings to broader populations, particularly in Western healthcare settings, is constrained by he geographical distribution of the included studies. Most trials were conducted in China, Iran, and Thailand, with limited representation from other regions. Furthermore, the generally small sample sizes and short follow-up durations may not fully capture the long-term treatment effects and safety profiles across diverse clinical populations and healthcare systems. Future multinational studies with larger sample sizes and extended follow-ups are warranted to validate these findings. (9) Formal economic evaluations were notably scarce among the included trials. Yang et al. (2024) provided the only robust cost-effectiveness analysis, showing significant cost reductions for phytotherapy. While these findings are promising, the singular nature of this evidence precludes definitive conclusions. Consequently, the economic implications discussed in Sect. "Implications" must be considered preliminary, underscoring the urgent need for prospective economic analyses in future trials [50].

### Implications

External Phytotherapy shows potential as a valuable adjunct in the management of diabetic foot ulcers (DFUs); translating these findings into clinical practice, however, requires careful consideration of the evidence base and its limitations.

Based on current evidence, cautious clinical applications may include: (1) considering topical phytotherapy as a supplementary intervention (not monotherapy) for Wagner Grade 1–2 DFUs; (2) prioritizing standardized plant extracts (e.g., Aloe vera gel, curcumin ointment) over compound formulations given superior efficacy (RR = 2.13 vs. 1.36; *p* = 0.009); and (3) maintaining vigilance for local adverse reactions despite favorable aggregate safety data. This aligns with broader safety principles, as plant-derived interventions require vigilance against rare tissue reactions [77].

The management of diabetic foot ulcers imposes substantial healthcare costs globally. The cost-effectiveness analysis by Yang et al. [13]. demonstrated significantly lower treatment expenditures with a novel phytotherapy regimen compared to conventional care. This economic advantage, combined with established efficacy, positions phytotherapy as a promising value-based strategy—potentially reducing costs while maintaining outcomes. The critical need for such cost-effective strategies is underscored by projections that the global burden of peripheral artery disease (PAD)—a key driver of DFUs—is expected to increase dramatically, affecting an estimated 360 million people by 2050 [78]. Future trials should integrate prospective economic evaluations. Similar value-based optimization has advanced oncology care through targeted approaches [79].

Our subgroup analysis demonstrated superior efficacy of plant extracts over compound formulations for complete ulcer healing (RR = 2.13 vs. 1.36; *p* = 0.009). This suggests that standardized extracts with defined bioactive components may provide more consistent therapeutic effects. However, limited head-to-head trials and heterogeneous outcome measures preclude identification of optimal species or extraction methods. Thus, while plant extracts are empirically favored, clinicians should prioritize agents with clinical bioactivity data until further comparative studies are conducted.

We discuss the assessment standards for the results. Despite the consistent utilization of image software in previous studies to measure ulcer areas, these studies overlook the consideration of ulcer depth [80]. A comprehensive evaluation model with a scoring system may be needed to assess ulcer depth, healing area dimensions (length and width), and overall healing progress [23]. Furthermore, a holistic approach should consider systemic factors, such as nutritional status, which is known to influence inflammatory processes and healing outcomes [81]. For vascular DFUs, Ankle Brachial Index (ABI) values can indicate the severity of vascular disease in the diabetic foot. Still, only one included study assessed ABI as an outcome measure [82]. External phytotherapy’s anti-inflammatory and antibacterial effects on DFUs also need further investigation [62]. The potential role of modulating the gut microbiome, an mechanism shared by other interventions such as intermittent fasting, represents a particularly interesting avenue for future research [83].​​ ​​Further investigation into mechanisms such as the PPARs/SHH-mitochondrial axis is warranted [84]. Broader outcome measures such as the ABI and internationally recognized pain relief and psychological assessment scales should also be considered for application. For neuropathic DFUs, monitoring of diabetic peripheral neuropathy, such as nerve conduction velocity measurement and neurological function score scales, is of great significance [57], particularly given psychological burdens in chronic wounds that impair adherence [85].

The substantial heterogeneity in phytotherapeutic formulations across included studies precludes specific recommendations regarding optimal drug selection for particular clinical scenarios. This limitation underscores the need for large-scale clinical trials and subsequent development of evidence-based clinical guidelines and expert consensus statements. Nevertheless, our findings provide valuable insights to inform clinical decision-making and guide future research directions. Notably, the superior therapeutic efficacy observed with plant extracts suggests a promising avenue for pharmacological development, potentially informing the design of novel therapeutic agents for DFUs’ management. This aligns with the broader potential of plant extracts, such as green coffee, to exert beneficial systemic metabolic effects [86], highlighting their value beyond topical application.​​​​It is imperative to note, however, that while these implications are promising, their generalizability is tempered by the predominantly Asian origin of the current evidence, underscoring the critical need for validation in Western populations and healthcare settings.​​.

## Conclusion

This GRADE-assessed meta-analysis of 20 randomized controlled trials suggests that external phytotherapy can be a beneficial adjunct to conventional diabetic foot ulcer care, potentially improving ulcer closure rates and wound area reduction, with a favorable safety profile. However, these findings should be interpreted considering the limitations, including heterogeneity and limited data for some outcomes. It is crucial to note that this is not a replacement for conventional therapy. These findings should be interpreted with caution due to the significant heterogeneity and limited data available for some outcomes. Future studies should explore long-term efficacy, optimal formulations, and mechanisms of action to strengthen clinical integration.

## Supplementary Information

Supplementary Material 1

Supplementary Material 2

Supplementary Material 3

Supplementary Material 4

Supplementary Material 5

Supplementary Material 6

Supplementary Material 7

## Data Availability

The article and its supplementary files contain the datasets that substantiate the findings mentioned in this paper.

## Abbreviations

ACAWD: Antimicrobial Calcium Alginate Wound Dressing
CHM: Chinese herbal medicine
CON: Control group
CPCF: Cortex phellodendri compound fluid
DFUs: Diabetic foot ulcers
FFHB: Fufang Huangbai Fluid
IN: Intervention group
KSF: Kangfuxin solution
RCTs: Randomized controlled trials
SUDE: Surgical debridement
SWT: Local debridement of necrotic tissue or callus, off-loading and dressing changes
WSA: Wound(ulcer)surface area
