# Supplementary material for: Efficacy and safety of external phytotherapy in diabetic foot ulcers: a GRADE-assessed systematic review and meta-analysis of randomized controlled trials
Source: Diabetol Metab Syndr. 2026 Jan 9;18:48. doi: 10.1186/s13098-025-02049-0 (PMC12879332; doi:10.1186/s13098-025-02049-0)
Supplement: Supplementary file 3 — Supplementary Material 3 [file 13098_2025_2049_MOESM3_ESM.docx]

**Supplementary file 3. Detailed Database Search Queries**

1.PUBMED:

(Chinese medicine[Title/Abstract] OR Chinese herbal[Title/Abstract] OR medicine herbal[Title/Abstract] OR herb[Title/Abstract] OR herbal[Title/Abstract] OR natural herb[Title/Abstract] OR natural products[Title/Abstract] OR medical plant[Title/Abstract] OR medicinal plant[Title/Abstract] OR plant[Title/Abstract] OR phytotherapy[Title/Abstract] OR phytomedicine[Title/Abstract] OR botany[Title/Abstract] OR botanicals[Title/Abstract]) AND (diabetic foot[Title/Abstract] OR diabetic foot ulcer[Title/Abstract] OR diabetic leg ulcer[Title/Abstract] OR foot ulcer[Title/Abstract] OR leg ulcer[Title/Abstract]) AND (clinical[Title/Abstract] OR RCT[Title/Abstract] OR random[Title/Abstract])

2.EMBASE

('chinese medicine':ti,ab,kw OR 'chinese herbal':ti,ab,kw OR 'medicine herbal':ti,ab,kw OR herb:ti,ab,kw OR herbal:ti,ab,kw OR 'natural herb':ti,ab,kw OR 'natural products':ti,ab,kw OR 'medical plant':ti,ab,kw OR 'medicinal plant':ti,ab,kw OR plant:ti,ab,kw OR phytotherapy:ti,ab,kw OR phytomedicine:ti,ab,kw OR botany:ti,ab,kw OR botanicals:ti,ab,kw) AND ('diabetic foot':ti,ab,kw OR 'diabetic foot ulcer':ti,ab,kw OR 'diabetic leg ulcer':ti,ab,kw OR 'foot ulcer':ti,ab,kw OR 'leg ulcer':ti,ab,kw) AND (clinical:ti,ab,kw OR rct:ti,ab,kw OR random:ti,ab,kw)

3.WOS

TS=((("Chinese medicine" OR "Chinese herbal" OR "medicine herbal" OR herb OR herbal OR "natural herb" OR "natural products" OR "medical plant" OR "medicinal plant" OR plant OR phytotherapy OR phytomedicine OR botany OR botanicals) AND ("diabetic foot" OR "diabetic foot ulcer" OR "diabetic leg ulcer" OR "foot ulcer" OR "leg ulcer") AND (clinical OR RCT OR random)))

4.Chorane

294 Trials matching Chinese medicine or Chinese herbal or medicine herbal or herb or herbal or natural herb or natural products or medical plant or medicinal plant or plant or phytotherapy or phytomedicine or botany or botanicals in Title Abstract Keyword AND diabetic foot or diabetic foot ulcer or diabetic leg ulcer or foot ulcer or leg ulcer in Title Abstract Keyword AND clinical or RCT or random in Title Abstract Keyword - (Word variations have been searched)

5. Additional Sources​​

Sources Included: ClinicalTrials.gov, WHO International Clinical Trials Registry Platform (ICTRP),Manual search of reference lists of included studies and relevant reviews, Contacted experts in the field for unpublished data.

Note: The search strategies for these sources were adapted to the functionality of each platform, primarily using simplified key terms (e.g., "diabetic foot ulcer" AND "herbal").

All searches were conducted from inception to April 30, 2024. No language restrictions were applied.
