# Supplementary material for: Efficacy and safety of external phytotherapy in diabetic foot ulcers: a GRADE-assessed systematic review and meta-analysis of randomized controlled trials
Source: Diabetol Metab Syndr. 2026 Jan 9;18:48. doi: 10.1186/s13098-025-02049-0 (PMC12879332; doi:10.1186/s13098-025-02049-0)
Supplement: Supplementary file 4 — Supplementary Material 4 [file 13098_2025_2049_MOESM4_ESM.docx]

**Supplementary file 4. Risk of Bias Assessment Method**

For studies that reported medians and interquartile ranges (IQR), we estimated the mean and SD using an online calculator (https://www.math.hkbu.edu.hk/~tongt/papers/median2mean.html) as described in Luo, et al. [2] and Wan, et al. [3]. However, when the data were not normally distributed, we eliminated the data rather than transformed it.

1.Luo D, Wan X, Liu J, Tong T. Optimally estimating the sample mean from the sample size, median, mid-range, and/or mid-quartile range. Stat Methods Med Res. 2018;27(6):1785-805. doi: 10.1177/0962280216669183.

2.Wan X, Wang W, Liu J, Tong T. Estimating the sample mean and standard deviation from the sample size, median, range and/or interquartile range. BMC Med Res Methodol. 2014;14:135. doi: 10.1186/1471-2288-14-135.
