# Supplementary material for: Efficacy and safety of external phytotherapy in diabetic foot ulcers: a GRADE-assessed systematic review and meta-analysis of randomized controlled trials
Source: Diabetol Metab Syndr. 2026 Jan 9;18:48. doi: 10.1186/s13098-025-02049-0 (PMC12879332; doi:10.1186/s13098-025-02049-0)
Supplement: Supplementary file 5 — Supplementary Material 5 [file 13098_2025_2049_MOESM5_ESM.docx]

**Supplementary File 5. Protocol for an Exploratory Analysis of Risk of Bias Using a Quantitative Scoring System​​**

1. Rationale and Objective​​

This document details the protocol for an exploratory analysis that was conducted in parallel with the standard Cochrane ROB2 assessment. The objective was to develop and apply a quantitative scoring system, derived directly from the official ROB2 judgments, to examine whether a more granular classification of methodological quality would influence the robustness of the primary meta-analysis findings.

2. Scoring Methodology​​

The scoring system was directly derived from the standard ROB2 tool's three-grade classification for each of the five assessment domains. A domain judged as 'Low risk' was assigned 3 points, a domain with 'Some concerns' was assigned 2 points, and a domain judged as 'High risk' was assigned 1 point. The overall risk of bias score for each study was calculated as the sum of the points from all five domains, yielding a total score ranging from 5 (indicating the highest risk of bias) to 15 (indicating the lowest risk of bias).

3. Classification Tiers​​

The continuous total score was categorized into five tiers to provide a gradient of methodological quality. A total score of 15 was classified as 'Low risk'. Scores between 13 and 14 with no high-risk domains were classified as 'Moderately low risk', while scores between 11 and 12 with no high-risk domains were classified as 'Moderately high risk'. A score between 6 and 13 with at least one high-risk domain was classified as 'High risk'; within this category, scores of 11-13 were specified as 'High risk' and scores below 11 (i.e., ≤10) were classified as 'Extremely high risk'.

4. Role in the Analysis​​

This predefined scoring system was used exclusively for an exploratory, secondary analysis to test the consistency of the conclusions generated by the standard ROB2 tool. The primary results and conclusions of the systematic review are based strictly on the standard Cochrane ROB2 classifications.

**Table 1. Comparison of Overall Risk of Bias Assessments: Standard ROB2 vs. Exploratory Scoring System​​**

| Study ID | Overall risk of bias(Standard ROB2) | Total score | Overall risk of bias |
| --- | --- | --- | --- |
| Argañaraz Aybar, Julio Nicolás.,2022 | Some concerns | 13 | Moderately Low |
| Chokpaisarn, Julalak.,2020 | Some concerns | 14 | Moderately Low |
| Du, J.C.,2011 | Some concerns | 12 | Moderately High |
| Fallah Huseini, H.,2021 | Low | 15 | Low |
| Fan, W.,2022 | Some concerns | 14 | Moderately Low |
| Huang, YY.,2021 | Some concerns | 14 | Moderately Low |
| Jacobs, A. M.,2008 | Low | 15 | Low |
| Li, F. L.,2011 | High | 13 | High |
| Li, S.,2011 | Some concerns | 14 | Moderately Low |
| Liu, Y. L.,2020 | Some concerns | 14 | Moderately Low |
| Najafian, Y.,2019 | Low | 15 | Low |
| Nasiri, M.,2015 | High | 13 | High |
| Romero-Cerecero, O.,2015 | Low | 15 | Low |
| Salahi, P.,2024 | Low | 15 | Low |
| Sanpinit, Sineenart.,2024 | Some concerns | 13 | Moderately Low |
| Tonaco, Luís A. B.,2018 | Low | 15 | Low |
| Xie, F,2012 | High | 10 | Extremely High |
| Xu, L.,2023 | Some concerns | 13 | Moderately Low |
| Yang, G.,2024 | Some concerns | 14 | Moderately Low |
| Zhan, H.,2021 | Some concerns | 14 | Moderately Low |
