# Supplementary material for: Efficacy and safety of external phytotherapy in diabetic foot ulcers: a GRADE-assessed systematic review and meta-analysis of randomized controlled trials
Source: Diabetol Metab Syndr. 2026 Jan 9;18:48. doi: 10.1186/s13098-025-02049-0 (PMC12879332; doi:10.1186/s13098-025-02049-0)
Supplement: Supplementary file 6 — Supplementary Material 6 [file 13098_2025_2049_MOESM6_ESM.docx]

**Table 1.** Basic characteristics of included studies

| Study ID | Country | NUM (IN, CON) | Gender (Male, Female) | Mean age | Inclusion Criteria | Duration of Diabetes (years) | Ulcer Duration  (months) | Ulcer grade | Study design | IN (dose, approach) | CON (dose, approach) | Intervention duration | Follow up | Outcomes |
| --- | --- | --- | --- | --- | --- | --- | --- | --- | --- | --- | --- | --- | --- | --- |
| Argañaraz Aybar, Julio Nicolás.,2022 | Iran | 12,10 | 16, 6 | - | Refractory DFU | - | - | Unclear | open-label randomized and controlled parallel clinical trial | SUDE + L.plantarum cultures solution | SUDE | 12 weeks | - | Adverse events, Microbial Analysis of DFU, Wound Area, Histopathology, Distribution and Numbers of Macrophages in DFUs, Neutrophils Functionality |
| Chokpaisarn, Julalak.,2020 | Thailand | 26, 25 | 11, 15 | 57 | DFU, grade 1-2 | 10.81±7.05 | 4.88±7.18（IN)，4.77±10.83(CON) | Grade 1-2 | multicenter, open-label, pre-test, and post-test randomized controlled trial | Quercus infectoria solution, topically applied once a day | Normal saline solution | 3 months or until wounds healed completely | - | Wound healing (percentage of wound area reduction, complete wound closure), adverse effects |
| Du, J.C.,2011 | China | 30,30 | 31, 29 | 64 | DFU | 10±3.4 | - | Grade 1-5 | randomized controlled trial | Basic treatment + traditional Chinese medicine composite film | Basic treatment +Vaseline gauze | Until ulcer complete closure or 24 weeks（168天） | - | Clinical efficacy (ulcer healing rate), average ulcer healing time |
| Fallah Huseini, H.,2021 | Iran | 29, 26 | 14,41 | 58.1±6.4（IN）,59.4±7.0(CON) | DFU, grade 1-2 | 15.2±7.4(IN), 13.3±7.2(CON) | - | Grade 1-2 | double-blind,placebo-controlled,randomized controlled trial | Standard treatment +topical T. polium ointment twice a day | Standard treatment +topical placebo ointment twice a day | 4 weeks | 8 weeks | Wound Healing Determined, Ulcer Recover, wound healing percentage, Blood Biochemical Test, adverse effects |
| Fan, W.,2022 | China | 44, 42 | 46, 40 | 70.95±7.70(IN),71.12±6.52(CON) | DFU, grade 2 -3 | - | - | Grade 2-3 | randomized controlled trial | Basic treatment+ EPTG+ Zizhu ointment） | Basic treatment + debridement and change of nano-silver medical antibacterial dressing | 12 weeks | - | Ulcer healing rate, area, depth, TCM symptom score, visual analogue scale, inflammatory factors, growth factors, adverse events, outcome events |
| Huang Y Y.,2021 | China | 122, 114 | 175, 61 | 57.0±10.9 | DFU, grade 1-2 | ≥10 years（61.0%） | 7．2±13.4 | Grade 1-2 | randomized controlled, evaluator-blinded phase 3 trial | ON101 applied topically twice daily | Hydrofiber; ConvaTecLtd, applied topically twice daily | 10 weeks | 16 weeks | Complete healing, Change in WSA, Incidence of patients with 50% reduction, Incidence of wound infection, Ulcer recurrence in WSA, HbA1c, adverse events |
| Jacobs, A. M.,2008 | America | 20, 20 | - | - | DFU, Wagner grade 1 or 2 | - | - | Grade 1-2 | A blinded study | Bensal HP with QRB7 ointment as adjunctive management | Silver sulfadiazine cream (SSC) as adjunctive management | 6 weeks | - | Combined wound diameter, Wound Culture Results, Wound Depth, adverse events |
| Li, F. L.,2011 | China | 27, 26 | 34, 28 | 54.1 ± 14.8(IN),46.2 ± 13.9(CON) | Diabetes with a diabetic skin ulcer | 8 (1-19) (IN), 6 (1-21(CON) | 0.72 ± 0.65 years (IN), 0.60 ± 0.41 years (CON) | Unclear | prospective, multi-center, randomized, single-blind, parallel controlled trial | "Hongyou Ointment" +"Shengji Powder" topically applied once a day | Mupirocin ointment, growth factor (bFGF，100 AU/cm^2^), Vaseline topically applied once a day | 4 weeks or until wound healing | - | Ulcer Healing Time, the expression levels of β-catenin, c-myc, and K6 proteins, adverse effects |
| Li, S.,2011 | China | 24, 24 | 31,17 | 60±13 | DFU, grade 1–3 | 9.8 ± 5.5 | ≥12 weeks | Grade 1-3 | multi-center, randomized, controlled, prospective, add-on clinical trial | SWT + TYO, every one to three days | SWT + saline gauze every one to three days | Until ulcer complete closure or 24 weeks | 24 weeks | Complete healing, ulcer improvement |
| Liu, Y. L.,2020 | China | 540, 180 | 397, 323 | 63.7±9.56 (IN),63.12±0.53 (CON) | DFU | 13.21±9.83(IN), 13.54±8.55(CON) | - | Grade 1-2 | multicenter clinical trial | CPCF | KSF | 4 weeks | short follow-up time | Wound area, Growth factor indexes, primary symptoms, secondary symptoms, treatment efficacy, Adverse events |
| Najafian, Y.,2019 | Iran | 20, 20 | 28, 12 | 61.5 ± 7.96(IN),57 ± 8.4(CON) | DFU, grade 1-2 | - | - | Grade 1-2 | double-blind randomized clinical trial study | Routine cares + Topical Aloe vera/ Plantago major gel (Plantavera gel) | Routine cares + Topical Placebo  gel | 4 weeks | - | Wound surface and depth, degree, color, drainage and surrounding tissues and scaling of the wound, the side effects |
| Nasiri, M.,2015 | Iran | 15，15 | 19, 11 | 53.8 ± 1.3(IN), 52.6 ± 9.13 (CON) | DFU, grade 1-2 | 12.73 ± 7.48 (IN), 14.93 ± 10.38 (CON) | 50.0 ± 28.65 (IN), 44.93 ± 30.37 (CON) | Grade 1-2 | double-blind randomized clinical trial study | Routine cares + Topical olive oil | Routine cares | 4 weeks | - | Ulcer surface area, Ulcer parameters scores, total ulcer status scores, Ulcer healing status, Adverse effects |
| Romero-Cerecero, O.,2015 | Mexico | 14, 16 | 17, 19 | 63±12.33 | DFU, grade 1-2 | ≥10 years（60%） | - | Grade 1-2 | Randomized, Controlled Pilot Study | A standardized extract (5%, cream formulation) of A. pichinchensis | Micronized silver sulfadiazine (1%) | 20 weeks-24weeks | - | Wound size reduction (percentage of wound area reduction), average time,lapse required for ulcers to heal, adverse events |
| Salahi, P.,2024 | Iran | 25, 25 | 36, 14 | 57.9 ± 9.88(IN),55.6 ± 10.44(CON) | DFU, grade 1-2 | 11.0 ± 8.18(IN), 12.8 ± 7.28 (CON) | 70.9 ± 89.23 days (IN), 60.8 ± 80.21 days (CON) | Grade 1-2 | randomized, placebo‐controlled, double‐blinded, parallel-group clinical trial | Standard care + Dermaheal ointment | Standard care + placebo | 4 weeks | - | DFU healing checklist (ulcer degree, ulcer color, ulcer peripheral tissues, and ulcer exudates,DFU size, DFU‐induced pain severity( a 0–10 Numerical Pain Rating Scale), Adverse effects |
| Sanpinit, Sineenart.,2024 | Thailand | 25, 25 | 34, 13 | 55.04 ± 1.87 (IN), 54.48 ± 1.60 (CON) | DFU, grade 1-2 | 6.04 ± 0.81 (IN), 10.28 ± 1.34 (CON) | 9.99 ± 1.79 months (IN), 4.88 ± 0.73 months (CON) | Grade 1-2 | Prospective, multicenter, open-label, randomized, controlled, and parallel-group study | Standard treatment + YaSP solution topically, 2 mL of the oil per cm^2^ of the wound area | Standard treatment | 12 weeks | - | Completely healed;Improved ulcer healing;Decreasing in ulcer area orWagner’s ulcer grade |
| Tonaco, Luís A. B.,2018 | Brazil | 27, 23 | 41，9 | - | diabetes 2 and neuropathic ulcers | - | - | Unclear | Double-Blind Randomized Pilot Study | Standard treatment +topical dressing formulated with 0.1% P1G10 | Standard treatment +hydrogel | 16 weeks | - | 100% or C 80% reduction of the initial total ulcer area;adverse effects |
| Xie, F.,2012 | China | 31, 31 | 45,17 | - | DFU | - | - | Unclear | Randomized controlled trial | Basic treatment + traditional Chinese medicine fumigation+ Shengji Yuhong cream | Basic treatment | 60 days | - | Clinical recovery rate, limb function, wound area, total effective rate, safety |
| Xu, L.,2023 | China | 40, 39 | - | 59.46±24.89 | DFU, grade 1-2 | - | 63.41±24.89 days | Grade 1-2 | Randomized controlled trial | Routine therapy +compound Huangbai  liquid fomentation once a day | Routine therapy +medical silver nanoparticles containing dressing once a day | 6 weeks | - | Ulcer area, ulcer depth, traditional Chinese medicine syndrome score, ABI, transcutaneous oxygen pressure, vascular endothelial growth factor,epidermal growth factor, advanced glycation end product, high-sensitivity C-reactive protein, adverse events |
| Yang, G.,2024 | China | 22, 20 | 29, 13 | 66.55 ± 11.959 (IN), 61.55 ± 10.283 (CON) | Diabetic foot infection (DFI) | - | - | Grade 2 | double arm study | FFHB + Basic treatment | ACAWD + Basic treatment | 2 weeks | 1 month | Negative rate of wound culture, change trend of minimum inhibitory concentration, infection control rate, Wound surface area, wound area healing rate, Pharmacoeconomic evaluations (Cost-effect ratio), Wound healing rate, adverse events |
| Zhan, H.,2021 | China | 25, 25 | 33, 17 | 65.1 ± 11.7 (IN); 63.4 ± 11.6 (CON) | DFU | - | - | Grade 2-4 | randomized controlled trial | Basic treatment + moist exposed burn ointment +Jinhuang powder | Basic treatment + moist exposed burn ointment | 1 month | - | effective rate, wound pain score |

Abbreviations: ABI: Ankle-brachial index; ACAWD: Antimicrobial Calcium Alginate Wound Dressing; CON: Control group; CPCF: Cortex phellodendri compound fluid; DFU: Diabetic foot ulcer; FFHB: Fufang Huangbai Fluid; IN: Intervention group; KSF, Kangfuxin solution; NUM, Number; SUDE, surgical debridement; SWT, local debridement of necrotic tissue or callus, off-loading, and dressing changes; TYO: Tangzu Yuyang Ointment.

^a^ Data not reported in the original publication, largely due to variations in reporting standards across studies from different time periods and regions.

The Hongyou ointment consists of Jiuyi Pellet (Gypsum Fibrosum: hydrargyrum oxydatum crudum = 9:1), Dong Pellet (main ingredient: minium), and Vaseline. Shengji Powder consists of Gypsum Fibrosuum, Resina Draconis, Resina Olibanum, Myrrh, and Borneolum syntheticum. It comprises Huangbai, Lianqiao (Forsythia suspensa), Jinyinhua (Lonicera japonica Thunb), Pugongying (Taraxacum mongolicum Handazz), and Wugong (Scolopendra). ON101 comprises two active pharmaceutical ingredients: PA-F4 from an extract of Plectranthus amboinicus and S1 from an extract of Centella Asiatica. Chinese medicine fumigation consisted of Astragalus 50 g, Sophora 20 g, Safflower 30 g, Angelica 30 g, Chuanxiong 15 g, Phellodendron phellodendron 20 g, Shuanghua 30 g, and Radix Paeoniae 20 g. Zizhu ointment, composed of Astragalus 9 g, comgrass 9 g, cinnabar 9 g, Dragon's blood 6 g, ejiao 6 g, borneol 3 g) Ruyi Jinhuang powder paste (Beijing Tongrentang,Jinhuang powder, proper amount of vinegar madeinto paste) (composed of trichosanthin, Cortex Phellodendri, rhubarb, turmeric, Angelica dahurica,purple Magnolia officinalis, tangerine peel, liquorice,Atractylodes Rhizoma, and Rhizoma Arisaematis)Bensal HP with QRB7 ointment, (formulation of benzoic acid, 6%; salicylic acid, 3%; and extract of Qrubra,3%)EPTG（Chinese medicine external therapeutic protocol of enriching pus for tissue growth）P1G10 (Latex of Vasconcellea cundinamarcensis)
