# Supplementary material for: Efficacy and safety of external phytotherapy in diabetic foot ulcers: a GRADE-assessed systematic review and meta-analysis of randomized controlled trials
Source: Diabetol Metab Syndr. 2026 Jan 9;18:48. doi: 10.1186/s13098-025-02049-0 (PMC12879332; doi:10.1186/s13098-025-02049-0)
Supplement: Supplementary file 7 — Supplementary Material 7 [file 13098_2025_2049_MOESM7_ESM.docx]

**Supplementary file 6. The funnel plot for each outcome**


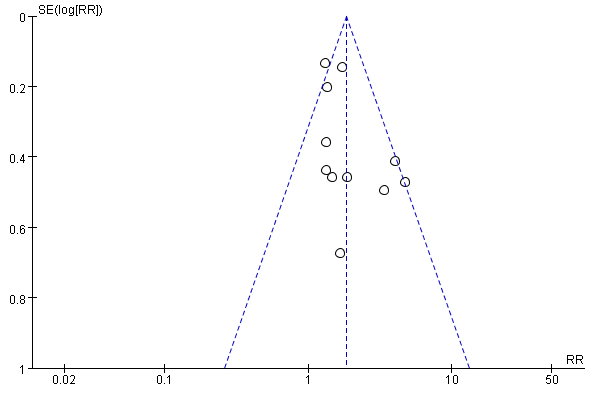


Figure S1. The funnel plot for complete ulcer healing


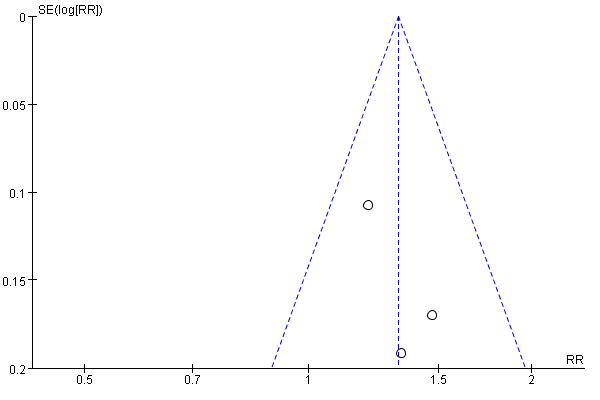


Figure S2. The funnel plot for improved ulcer condition


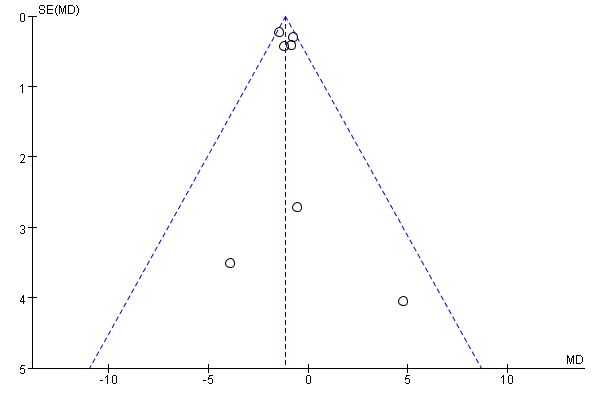


Figure S3. The funnel plot for Area of ulcers.


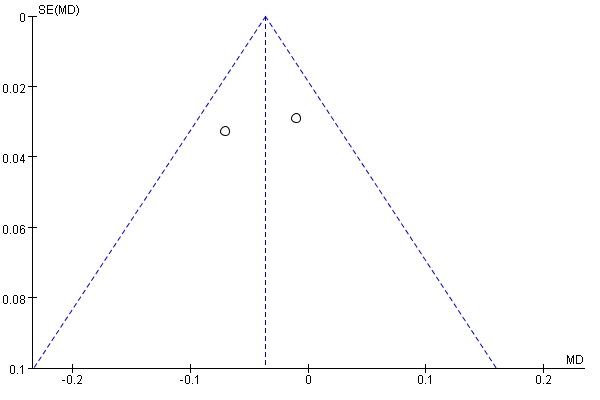


Figure S4. The funnel plot for ulcer depth


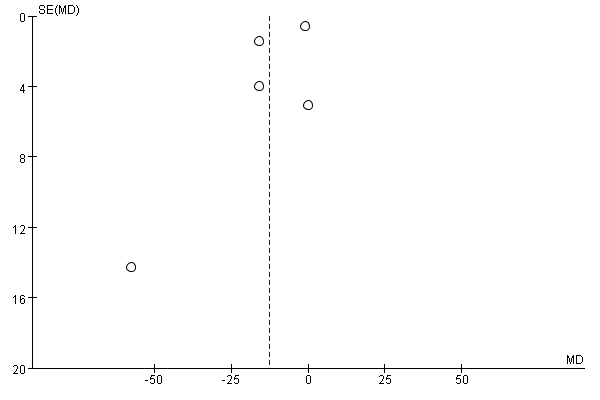


Figure S5. The funnel plot for percentage reduction in ulcers.


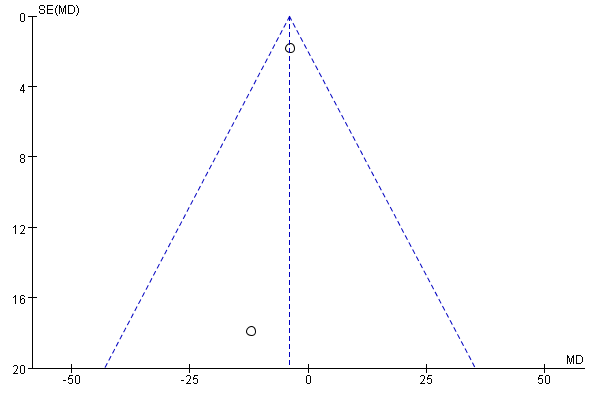


Figure S6. The funnel plot for ulcer healing time


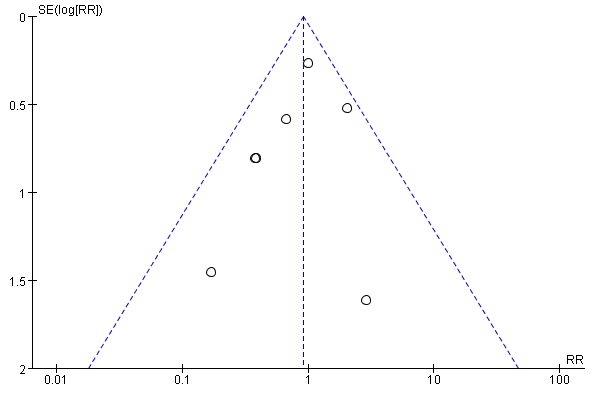


Figure S7. The funnel plot for adverse events
